# Virtual social grooming in macaques and its psychophysiological effects

**DOI:** 10.1038/s41598-024-62638-3

**Published:** 2024-05-22

**Authors:** Eloïse Disarbois, Jean-René Duhamel

**Affiliations:** Institut des Sciences Cognitives Marc Jeannerod, CNRS, Bron, France

**Keywords:** Social, Grooming, Touch, Macaques, Heart rate, Body schema, Animal behaviour, Social behaviour

## Abstract

Allogrooming is a widespread, pervasive activity among non-human primates. Besides its hygienic function, it is thought to be instrumental in maintaining social bonds and establishing hierarchical structures within groups. However, the question arises as to whether the physiological and social benefits derived from social touch stem directly from body stimulation, or whether other mechanisms come into play. We address this question by analyzing an elaborate social behavior that we observed in two adult male macaques. This behavior demonstrates the existence of a persistent motivation to interact through a form of simulated grooming, as the animals were housed in adjacent enclosures separated by a glass panel preventing direct tactile contact. We find that such virtual grooming produces similar physiological sensations and social effects as allogrooming. We suggest that this behavior engages affective and reward brain circuits to the same extent as real social touch, and that this is probably achieved through high level processes similar to those involved in bodily illusions or synaesthetic phenomena previously described in humans. This observation reveals the unsuspected capacity of non-human primates to invent alternative, quasi-symbolic strategies to obtain effects similar to those provided by direct bodily interaction, which are so important for maintaining social bonds.

## Introduction

Macaques spend a considerable amount of their time grooming each other. Besides its hygienic function, grooming is considered instrumental in maintaining social bonds and establishing hierarchical structures within groups^1^, and has been put forward as a social commodity, that can be reciprocated^2^ or exchanged for other benefits, such as social support in the face of conflicts^3^. The main hypothesis behind this view is that engaging in allogrooming is relaxing and can alleviate stress. This is supported by physiological evidence in various species of macaque monkeys, such as heart rate reduction^4,5^ and lower blood cortisol levels^6^ in groomed individuals, and lower feacal glucocorticoids concentrations in groomers^7^. Behavioral indicators also point at a reduction of anxiety through allogrooming: reduction in self-directed behaviors and displacement activities have been reported following the giving^8–11^, and receiving of grooming^12^. These overall benefits are thought to originate in the activation of C-tactile afferents, triggering the release of key neurotransmitters, such as oxytocin, as well as endorphins^13,14^ during grooming. However, the question arises as to whether the physiological and social benefits derived from grooming stem directly from body stimulation, or whether other mechanisms come into play. In laboratory settings, monkeys sometimes need to be single-housed and are therefore prevented from performing this core social behavior, despite living in colony rooms and having visual access to congeners. Here, we report a recurrent behavior (hereafter: “glassgrooming”) between two laboratory macaques that seems to mimic grooming through a glass panel separating their cages. We investigated how this behavior manifested itself and was reinforced and whether it induced physiological changes similar to actual allogrooming.

## Results

**Figure 1:**
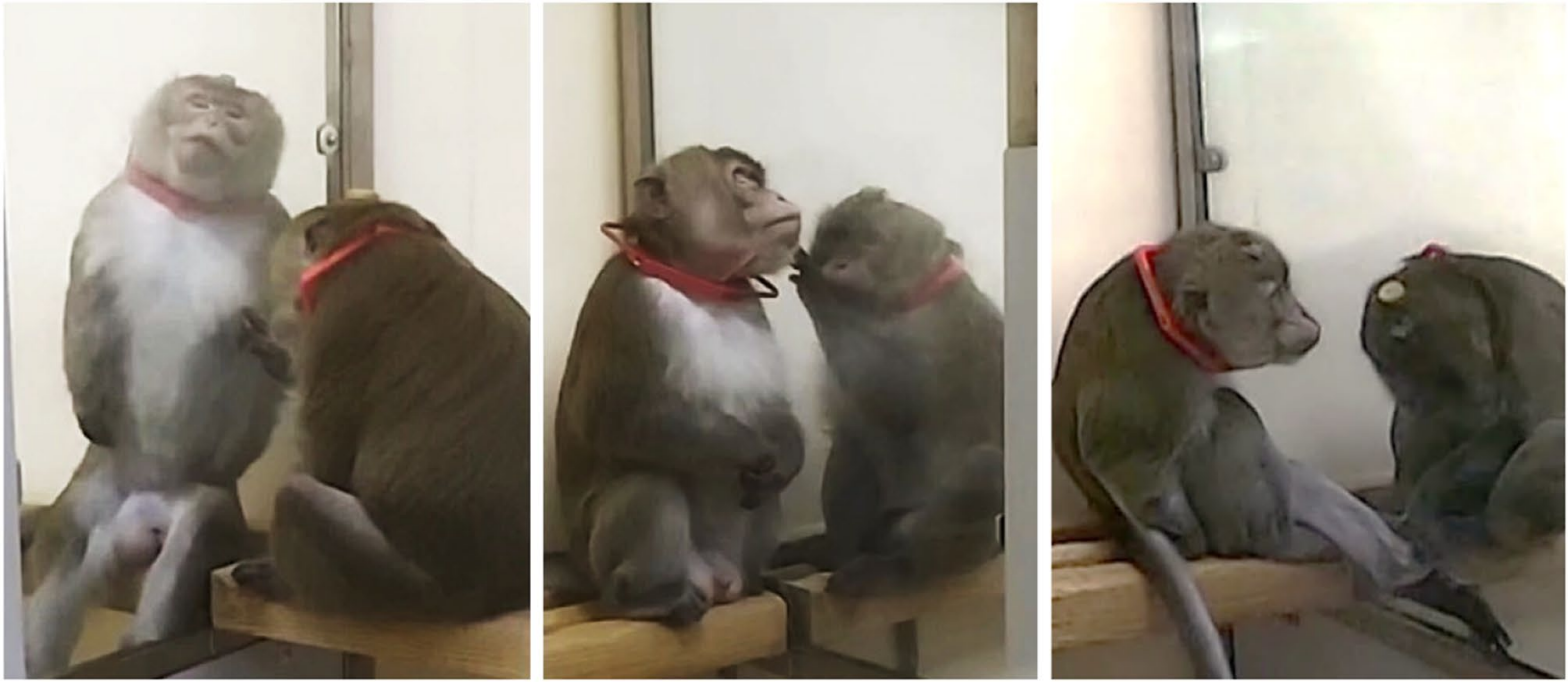
Pictures of the groomee (left) and the groomer (right) engaging in virtual grooming (“glassgrooming”) though the glass, with the groomee presenting body parts.

**Figure 2:**
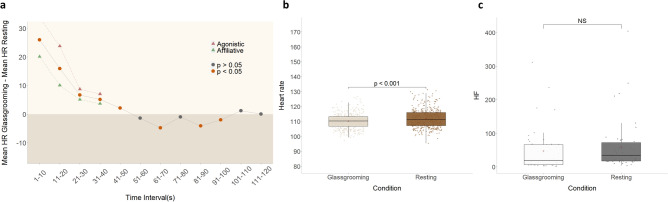
Cardiac measures during glassgrooming. (**a**) Difference in the groomee’s heart rate (HR) values during Glassgrooming and Resting in 10 s intervals from the start of the bouts (time = 0). Points represent the mean difference for the given interval, and are colored orange if for said difference, *p* < 0.05 (Wilcoxon rank-sum test). *Red triangles* = Difference between mean HR for glassgrooming started with an agonistic interaction and mean HR for resting; *green triangles* = difference between mean HR for glassgrooming started with an affiliative interaction and mean HR for resting. (**b**) Boxplot of HR during glassgrooming and resting for the interval 50–120 s (Wilcoxon rank-sum test). (**c**) Boxplot of HF during glassgrooming and resting for the interval 50–120 s (Wilcoxon rank-sum test).

Over the course of 55 video recording sessions, spanning a period of 16 months, we identified 10 glassgrooming interactions, lasting on average 473.4 s (min = 37 s, max = 802 s; sd = 292.3). These interactions involved active engagement of a fixed “groomee”, and a “groomer”, with the groomee showing body parts he would like to be groomed, and the groomer performing grooming-like sweeping gestures that flexibly adapt to the other’s postural changes (Fig. 1 and Supplementary Video S1). Such interactions were preceded by a behavioral sequence lasting on average 19.7 s (min = 4 s, max = 32 s, sd = 10.47), and triggered by either one of the monkeys, or both simultaneously (Supplementary Video S2). This sequence started with affiliative (70%) or agonistic (30%) behaviors, but always ended with mutual affiliative behaviors before the beginning of glassgrooming. Both monkeys showed agitated behavior whether the encounter was affiliative or agonistic, with behaviors such as genital presentations, lipsmacking, jumping and tapping the glass panel. In 2 sessions, external conflict in which the groomer was engaged and the groomee provided support preceded glassgrooming. In the 3 cases in which the groomee started the interaction with aggression, the groomer was resting behind the glass and not looking at or engaging with the other monkey. We did not observe other situations in which the groomee started the interaction with aggression (for instance, the groomee resting behind the glass while the groomer approached). In one session, the groomee began the interaction by approaching the glass while the groomer rested behind it, the groomer responded by showing aggressive behaviors, followed by the usual sequence of mutual affiliative displays and then glassgrooming. Therefore, meetings between the monkeys at the glass interface could have outcomes of opposite valence, which both parties could influence. To assess physiological changes associated with glassgrooming, we recorded heart rate in the groomed animal, in 7 sessions. Our analysis reveals that heart rate during glassgrooming is lower than in control resting periods (Wilcoxon rank-sum test, z = − 3.5, *p* = 0.0004195) (Fig. 2). Heart-rate variability measures showed no significant difference between conditions.

### Experimenter-groomee glassgrooming

**Figure 3:**
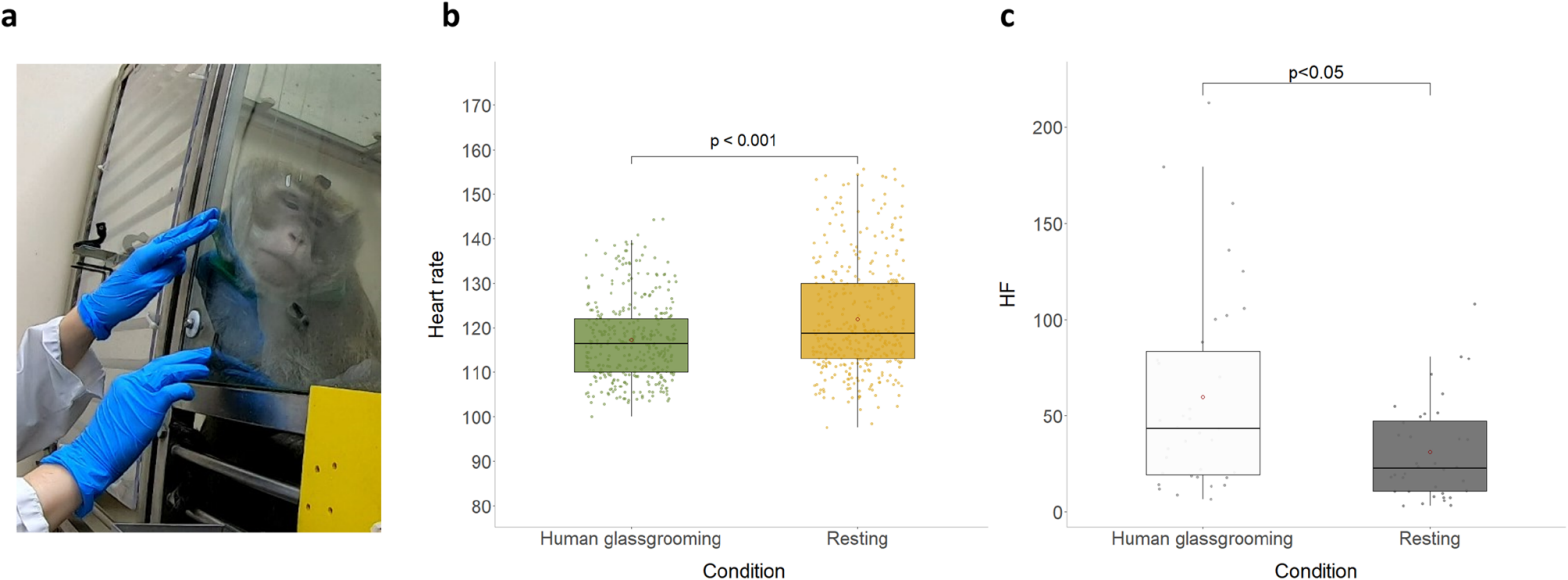
Human-groomee interactions. (**a**) Picture of the groomee being groomed through the glass by the experimenter. (**b**) Boxplot of HR during human glassgrooming and resting (Wilcoxon rank-sum test). (**c**) Boxplot of HF during human glassgrooming and resting. (Wilcoxon rank-sum test).

We successfully managed to transfer the behavior to the experimenter-groomee interaction, on several occasions, indicating this behavior is generalizable (Supplementary Video S4). We collected heart rate data in 4 of these interactions and 4 control resting periods. We show that heart rate is significantly lower during human glassgrooming than in matching resting periods. (Wilcoxon rank-sum test, z = − 4.75, *p* = 0.000001953). Contrary to glassgrooming between groomer and groomee, experimenter glassgrooming showed significantly higher HF values during human glassgrooming (Wilcoxon rank-sum test, z = − 2.53, *p* = 0.01135) (Fig. 3).

### Dominance test

In order to characterize the dominance relationship of the pair, we performed 4 sessions of a food-grab test. The groomee retrieved the food in 20 out of 23 trials (~ 87%). Interestingly, two out of these four tests induced glassgrooming (Supplementary Video S3).

## Discussion

We report the existence of a persistent, until now undescribed form of spontaneous social interaction between two laboratory macaque monkeys. Its triggering in situations of competition and conflict, but also affiliative contexts, indicates that it helps regulate tensions amongst the pair, and could strengthen bonds and foster support in the face of external conflict. Furthermore, we show that during glassgrooming, the groomee’s heart rate is lower than in control conditions. Therefore, it seems that this interaction uses the social codes of allogrooming, adapts them to the restricted social life and environment constrained by laboratory settings, and goes so far as to produce physiological effects found to be present in allogrooming^4,5^, hinting at a relaxed state of the groomee. HF measures suggest an accompanying vagal tone in the human-groomee interactions which was not significant for monkey glassgrooming. This may be explained by the ultra-short segment durations used here, which are not the standard for HRV measures^15,16^, and the rarity of our data did not allow to investigate this further. Considering the complex social dynamics that seem to underlie this behavior, its high degree of flexibility and generalization to a human groomer with the same associated reduction in heart rate, we argue that it does not qualify as stereotypical behavior. Instead, we explain this behavior’s emergence by the fact that the glass interface is a socially charged region with high incertitude regarding meeting outcomes and thus, glassgrooming arose as a tension-reduction mechanism to overcome the evolving, unstable status of the area. For this to be the case, we argue that this behavior must engage socio-affective and reward brain circuits and neuromodulators (especially oxytocin, serotonin and dopamine) to the same extent as real social touch^17^. Indeed, although the monkeys seemingly derive no immediate benefit^18^ from this behavior, the fact that it occurs regularly over months and sometimes lasts more than 10 minutes, suggests that it is reinforced by its positive social and physiological effects, during the interaction, and on a larger timescale. We further suggest that these effects are mediated by high-level multisensory mechanisms previously described in humans. The perception of the body and immediate peripersonal space is indeed highly plastic and can give rise to striking illusions, such as the integration of an artificial limb into the body schema^19,20^ or the experience of tactile sensations from a visual stimulus as seen in mirror-touch synesthesia^21,22^. Integrative circuits in the parietal and frontal cortical regions receiving convergent inputs from visual, somatosensory and other modalities^23,24^ are generally considered to be the neural substrate of bodily self-awareness^25^. We could therefore hypothesize that glassgrooming induces pleasant sensations in the groomed animal through the activation of these neural ensembles by vision. As to the groomer’s participation in this social interaction, one could suggest that vicarious processes could be at play, and engage similar mechanisms and brain circuits that have been found to support empathic responses to both painful and rewarding stimuli^26,27^, and the still poorly understood mechanisms involved in giving grooming^28^. Alternatively, we could argue that what appears to be a calming effect stems from mechanisms that do not involve the feeling of touch. Through the association of tactile sensations and a specific individual, which could in our case have occurred during the pair’s youth as they had experienced shared group-housing at that time, the monkeys may be seeking the interaction for itself. However, it is worth noting that the subjects have not been in direct contact for several years, therefore if such a behavioral association underlies this behavior, it would be surprisingly resilient, highlighting the untiring social nature of these primates. The ability of the groomee to show this behavior and associated effects when interacting with the experimenter hints at the fact that an associated bond might be dispensable, and that the physiological effects might arise on the sole basis of allogrooming being an inherent part of macaque social behavior. Therefore, another possibility is that the visual stimulation previously paired, during past real-life grooming, with C-afferent stimulation, produced, through a process akin to pattern completion, the same pleasant sensations and bodily states. Such effects could be mediated through multimodal limbic structures such as the insula and amygdala, that are involved in the processing of pleasant touch in humans and monkeys^29–33^.

To sum up, this study points at the possibility of additional mechanisms involved in making social touch beneficial, besides direct body stimulation, and of high-level mechanisms that sustain this adapted form of social behavior. The genesis and maintenance of this singular expression of affiliative behavior sheds new light on the extent to which non-human primates perceive and respond to each other's need for social contact.

## Materials and methods

### Subjects

All procedures conducted on the animals conformed to European and National Institutes of Health Guidelines for the Care and Use of Laboratory Animals, and complied with ARRIVE guidelines. They were approved by the Regional Ethics Committee (CELYNE). The subjects were two adult male fascicularis macaques, aged 13 at the beginning of the study. They were housed individually, in adjacent cages separated by a 8 mm thick glass panel from which they could see each other and the rest of the colony room. The two monkeys arrived in the lab juvelines from a large breeding colony and were initially housed in a 4-animal, male-only group, until puberty, when permanent conflicts made their separation necessary around 8 years before this study. Since then, groomer and groomee have only been single-housed, but in proximity.

### Glass interface

In order to ensure that vibrations transmitted through the glass couldn’t evoke somatosensory stimulation, we placed a piezoelectric sensor on one side of the glass panel and compared the electrical signal output between touching the sensor directly, and from behind the glass panel as during glassgrooming. When applying equal or greater pressure than the groomer and experimenter during glassgrooming, no vibrations were recorded by the sensor on the other side of the glass panel.

### Data collection

Videos were recorded with 2 GoPro Hero 7 cameras that were placed in front of each of the monkeys’ cages, behind a protective plexiglass panel. External batteries were used to permit long video recordings. The first set of sessions were video-only, and were recorded between February and April 2022. The second set of sessions included telemetric recordings of cardiac data and was collected between the months of January and July 2023. Sessions were recorded in the mornings and afternoons, and lasted up to 4 h. The videos were labelled with BORIS 8.8.1^34^. We also collected glassgrooming data with the experimenter as a groomer. The glass interface involved in this interaction was located in the front of the groomee’s cage, and was not the same as the one in which the two monkeys perform glassgrooming, despite having the same properties. The monkey would sit on the platform behind the glass interface and voluntarily interact with the experimenter.

### Telemetry device implantation

The groomee was implanted with a PhysioTel® Digital L04 telemetry device (Data Sciences International, DSI, St Paul MN, USA). The animal was pre-anesthetized with ketamine and medetomidine and subsequently maintained on isoflurane (1–2%) throughout the surgical procedure. Vital signs, including heart rate, respiratory rate, and blood oxygen saturation, were continuously monitored. The telemetric device was placed on the lower abdomen through a small incision, and its sensors subcutaneously tunneled to the thoracic region. The subject received postoperative analgesia, antibiotic, anti-inflammatory medication prior to and for a few days after surgery. He was closely monitored during the recovery period to ensure proper and comfortable wound healing. The implant was activated at the beginning of each session by placing a magnet in front of it, which the monkey was trained and rewarded for. Two transceivers were placed above and on the side of the cage of the subject, and collected signal from the implant which was transmitted to a dedicated telemetry computer through the communication link controller. The two animals had previously undergone a separate cranial implant surgical procedure for purposes unrelated to the current study.

### Data processing and analysis

A timestamped event was sent to the DSI software Ponemah (v6.51) at the beginning and end of each session to allow the synchronization of video and physiological data with precision to the second. At the end of each session, heart rate data was processed from raw signal as the reciprocal of the RR intervals and exported with the Ponemah software as their harmonic mean over 1-s periods. Heart rate values that were 3 standard deviations above the mean in each condition were considered outliers, and removed from analysis. Videos were coded in BORIS and timestamps aligned with telemetry data using a custom Matlab R2018b^35^ code. Analyses were performed using R and R studio^36,37^. To serve as control periods, resting events where the monkey was located at the same position than during glassgrooming were selected. Since the approach and presence of other individuals, and anxiety has been shown to modulate heart rate^4^ and especially considering our hypothesis regarding the social importance of the glass interface, we restricted our analyses to resting periods when the groomer was also resting nearby. Since the glassgrooming bouts were preceded by energetic behavioral displays and arousing social situations, heart rate tended to greatly rise, and remain high for the first part of the glassgrooming bout. We did not find matching situations that would allow control periods to integrate this aspect in our analysis, and therefore, using the glassgrooming bouts as a whole for comparison with resting skewed results. Tracing heart rate difference in 10 s bins did show this higher starting point for glassgrooming, and rapid decrease compared to resting periods (see Fig. 2). Using the first bin that didn’t show a difference (Wilcoxon rank-sum test) between resting and glassgrooming values as the starting boundary for heart rate comparison, allowed to reduce the effect of the pre-glassgrooming sequence on the overall heart rate during glassgrooming. We bounded the analysis window based on the shortest glassgrooming bout of 120 s to control for the effect of the duration of sitting still on HR, excluding one glassgrooming and one matching control resting bout under 60 s for the results shown in Fig. 2b (n = 6 glassgrooming, and n = 6 control resting bouts). Note that the co-occurrence of autogrooming by the groomee was repeatedly observed during glassgrooming. Autogrooming tended to generate movements and therefore raise the heart rate, which is consistent with previous reports of heart rate data for macaques’ behavior^5^.

For the human-experimenter interactions, we collected cardiac data in 4 human glassgrooming bouts, and 4 matching resting periods. They were processed in the same manner as the groomer-groomee data. We made sure that the behaviors preceding them matched in intensity. We trimmed every bout to the shortest measured one, which was of 92 s, to control for the effect of the duration of sitting still on heart rate.

Heart rate variability measures were extracted from the fast-Fourier-transformed raw signal in 10 s windows. The high frequency power band, HF, was used as an indicator of vagal tone. The upper and lower limits of this band were set as 0.15–0.5 Hz based on monkey studies^38,39^.

### Dominance test

To study dominance, we performed 4 sessions of a food-grab test^40^. We used a homemade pole to place a piece of fruit or peanut (we confirmed the monkey’s liking of the proposed food and desensitization to the pole prior to the start of the test) above the glass separating the cages of the two monkeys. Each trial began by making the monkeys come to the front of their cages so that they would be at equal distance to the food that was subsequently provided thanks to the pole. Grabbing the reward demanded to climb up to the roof of the cage before the other contestant did, and reaching the food by passing the arm through the cage’s wire. Trials were video recorded with two GoPro Hero 7 cameras, and live-annotated by a second experimenter.

### Supplementary Information

Supplementary Video 1.

Supplementary Video 2.

Supplementary Video 3.

Supplementary Video 4.

## Data Availability

The datasets generated during the current study are available from the corresponding author on reasonable request.
